# Effects of Dietary Supplementation with Epigallocatechin Gallate on Meat Quality and Muscle Antioxidant Capacity of Broilers Subjected to Acute Heat Stress

**DOI:** 10.3390/ani11113296

**Published:** 2021-11-18

**Authors:** Fei Zhao, Xiaocheng Wang, Yang Li, Xingyong Chen, Zhaoyu Geng, Cheng Zhang

**Affiliations:** Department of Animal Science, College of Animal Science and Technology, Anhui Agricultural University, Hefei 230036, China; zhaofei550516@163.com (F.Z.); wxc964295363@163.com (X.W.); liyangdxs2021@163.com (Y.L.); chenxingyong@ahau.edu.cn (X.C.); gzy@ahau.edu.cn (Z.G.)

**Keywords:** acute heat stress, epigallocatechin gallate, meat quality, antioxidant capacity, broiler

## Abstract

**Simple Summary:**

Broilers are readily affected by acute heat stress (AHS), and the development of intensive and high-density management and the occurrence of high temperatures during the summer exacerbate this problem. AHS has undesirable effects on animal immunity, meat quality, antioxidant capacity, and welfare of broilers, which can be alleviated by nutrition regulation. Epigallocatechin gallate (EGCG) has been found to reduce the damage of AHS on growth performance, antioxidant capacity, and the expression of nuclear factor erythroid 2-related 2 (*Nrf2*) in liver and jejunum. However, there are few reports on the effect and mechanism of action of EGCG on meat quality and antioxidant function in broilers under AHS. We demonstrated that EGCG protects against AHS-impaired meat quality by improving muscle antioxidant capacity, which seems to be associated with the activation of the *Nrf2* signaling pathway. Moreover, these findings suggested that EGCG could be an effective additive to improve meat quality and muscle redox balance by regulating the *Nrf2* signaling pathway.

**Abstract:**

This study evaluated epigallocatechin gallate’s (EGCG’s, 400 mg/kg) effect on meat quality and muscle antioxidant status of broilers under acute heat stress (AHS). A total of 144 21-day-old male Huainan partridge chickens were randomly allocated to the EGCG-free group (12 replicates) and the EGCG group (6 replicates). On day 94, the EGCG-free group was divided into the control group (CON) and the AHS group, and then AHS group and EGCG group (identified as AHS + EGCG group) were treated with AHS (33 ± 1 °C for 12 h). AHS increased (*p* < 0.05) L*_24h_, drip loss, muscle lactic acid, malondialdehyde (MDA) contents, and kelch-like ECH-associated protein 1 (*Keap1*) mRNA level, and decreased (*p* < 0.05) eviscerated percentage, pH_24h_, a*, muscle total superoxide dismutase (T-SOD) activity, the ratio of T-SOD/MDA and glutathione peroxidase /MDA, glycogen content, and nuclear factor erythroid 2-related 2 (*Nrf2*), catalase (*CAT*), NAD(P)H/quinone dehydrogenase 1 (*NQO1*) mRNA levels. The AHS + EGCG group exhibited lower (*p* < 0.05) L*_24h_, drip loss, muscle lactic acid, MDA contents and *Keap1* mRNA level, and greater (*p* < 0.05) eviscerated percentage, pH_24h_, a*, muscle T-SOD activity, the ratio of T-SOD/MDA, *Nrf2*, and *NQO1* mRNA levels compared with the AHS group. In conclusion, EGCG protects against AHS-impaired meat quality by improving muscle antioxidant capacity, which seems to be associated with the activation of the *Nrf2* signaling pathway.

## 1. Introduction

Broilers are readily affected by acute heat stress (AHS), and the development of intensive and high-density management and the occurrence of high temperatures during the summer exacerbates this problem [1]. A previous study has reported that AHS has undesirable effects on animal immunity, antioxidant capacity, and meat quality, inducing substantial economic consequences in the broiler industry [2,3,4,5]. Hence, the harmful effects of AHS on birds have become a matter of concern for the poultry industry, and domestic and foreign scholars have sought exploration of efficient methods to attenuate the stress response.

A previous study reported that a hot environment negatively affects the meat quality and even results in PSE (pale, soft, exudative) meat [6]. Moreover, AHS can increase the contents of hydrogen peroxide and superoxide free radicals. The production of excessive free radicals negatively affects the redox balance of the body [7,8]. Dietary supplements with antioxidant properties, such as resveratrol [6], curcumin [8], and glutamine [9] can mitigate the negative effects of heat stress (HS) and oxidative stress to improve the meat quality and muscle antioxidant capacity of chickens. Epigallocatechin gallate (EGCG) is a polyphenolic component of green tea and has many potential health effects, including antioxidant, anti-carcinogenic, hypocholesterolemic, and cardioprotective epigenetic activities [10]. Wang et al. reported that EGCG partially improved the antioxidant system in vanadium-challenged hens [11]. Moreover, previous studies of broilers subjected to cyclic chronic HS found that dietary EGCG supplementation can effectively improve growth performance, antioxidant capacity, and the expression of nuclear factor erythroid 2-related 2 (*Nrf2*) in liver and jejunum, kidney, and ovary [12,13,14]. Nevertheless, there exists no report on the effect and mechanism of action of EGCG on meat quality and antioxidant function in broilers under AHS. Therefore, this study aimed to explore the potential protective effect of EGCG on meat quality and redox balance in the breast muscle of broilers subjected to AHS and reveal its mechanism based on the Nrf2 signaling pathway.

## 2. Methods and Materials

### 2.1. Animals, Diets, and Management

This study’s experimental protocol was approved by the ethics committee of Anhui Agricultural University, Hefei, P.R. China (approval code: 2020357). A total of 144 male Huainan partridge broilers (21 days old, obtained from the experimental base of Anhui Agricultural University) with similar body weights (700 ± 20 g) were divided into two dietary treatment groups; the EGCG-free group included 12 replicates of eight birds each, and the EGCG group included six replicates of eight birds each. All birds were housed in stainless steel pens (1.2 × 1.0 m^2^). Each pen has a pan feeder with a 25 cm diameter and 3 nipple drinkers. The birds of the EGCG-free group were fed a basal diet, and the birds of the EGCG group were fed a basal diet with 400 mg EGCG (Sigma) per kg diet. Song et al. [12] showed that the additive levels of EGCG at 300 and 600 mg/kg can alleviate the oxidative damage of intestinal tract of broilers caused by chronic heat stress. Therefore, the effects of different doses (0, 200, 400, 600, and 800 mg/kg) of EGCG on the antioxidant capacity of broilers were explored in the early stage of this study, and it was found that the supplemental level of 400 mg/kg had significant improvement effect. Accordingly, an additive dose of EGCG at 400 mg/kg was used in this study. The basal diet (Table 1) was formulated according to the feeding standard of chickens of China (Criterion Code: NY/T 33-2004). All diets were provided in mash form, and the birds had free access to feed and water. All birds were raised in the climatized rooms at thermo-neutral conditions (according to Huainan partridge broiler guidelines) until 93 days of age. Briefly, room temperature was maintained at 22 ± 1 °C, the relative humidity was around 60%, and artificial light (25 lux) was provided 23 h per day by using LED lights. At 94 days of age, the EGCG-free group was divided into two equal groups: the control group (CON) and the AHS group. The birds of the CON group were housed at thermo-neutral conditions (22 ± 1 °C), while the birds of the AHS group and EGCG group (identified as AHS + EGCG group) were exposed to AHS treatment at 33 ± 1 °C for 12 h from 17:00 to 5:00, and the relative humidity remained at around 65% before sampling. As we have found that EGCG supplementation could improve the antioxidant function of broilers housed at thermo-neutral conditions (unpublished data), we hypothesized that EGCG may improve the meat quality of acute heat-stressed broilers through improving the muscle antioxidant capacity. Therefore, this study was conducted using the fractional factorial design.

### 2.2. Samples and Collection

Six replicates may be too few for the determination of carcass traits, meat quality, and antioxidant capacity. Therefore, after heat exposure, 8 birds from each group (one or two birds per replicate) were randomly chosen, weighed, and sacrificed by cervical dislocation. The eviscerated carcass weight and semi-eviscerated carcass weight were measured as previously described [15]. Breast muscle (pectoralis major) samples were then collected. One portion of each sample was stored at 4 °C and used for meat quality determination. The other portion was quickly frozen in liquid nitrogen and stored at −80 °C for further antioxidant enzyme activity assaying, metabolite measurement, and RT-PCR analysis.

### 2.3. Meat Quality Analysis

The right side of the breast muscle (*n* = 8) at 45 min and 24 h postmortem was used for determining meat color (L* = lightness, b* = yellowness, and a* = redness) using an automatic chroma meter (CR-400, Minolta Camera, Osaka, Japan) equipped with D65 illuminator, 10° observer, and an 8 mm diameter aperture. The final value for meat color was the average of three readings. The pH was measured at 45 min and 24 h postmortem using a handheld digital pH meter (PHSJ-5; Shanghai INESA Scientific Instrument Co. Ltd., Shanghai, China), which was calibrated with pH 4.6 and 7.0 buffer solutions. The measurement values of three different areas were recorded and averaged according to national measurement standards (Criterion Code: NY-T/1333-2007). Cooking loss was determined as previously described [6], the breast muscle sample of about 20 g was taken and placed in an airtight bag, heated to 75 °C in a water bath of 80 °C, cooled and surface water wiped off, and the weight difference before and after cooking was used to determine cooking damage. The cooled samples were cut vertically into long strips (2 × 1 × 1 cm), and shear force was determined using a texture analyzer (C-LM4-23-68910, Harbin, China). The left side of the breast muscle was used for the measurement of drip loss, as previously described [6]. A breast muscle sample of about 20 g was hung in an airtight container with a hook so that it did not touch the container wall, and then stored for 24 h at 4 °C. After storage, any moisture on the surface of the meat sample was removed with filter paper and weight of total water loss was determined. The difference in weight was used to determine drip loss.

### 2.4. Metabolite Content and Enzyme Activity Assay

Muscle tissue (*n* = 8) was homogenized in ice-chilled 0.9% sodium chloride solution, and the homogenate was centrifuged at 3500× *g* for 8 min at 4 °C to obtain the supernatant which was used to measure the activities of catalase (CAT), total superoxide dismutase (T-SOD), and glutathione peroxidase (GSH-Px), as well as the contents of malondialdehyde (MDA), protein carbonyl (PC), glycogen, and lactic acid at appropriate dilutions. The protein content of the supernatant was determined by the Bradford method using bovine serum albumin as the standard as previous described [16]. The above measurements were determined with commercially available kits (Nanjing Jiancheng Institute of Bioengineering, Nanjing, Jiangsu, China) according to the corresponding instructions. In addition, the usefulness of these kits has been shown for broilers in previous studies [6,17].

### 2.5. Real-Time PCR Analysis

Total RNA was extracted from muscle samples (*n* = 8) using TRIzol (Yeasen, Shanghai, China) and then reverse-transcribed into cDNA (Yeasen, Shanghai, China) following the protocol of the manufacturer. The mRNA levels of the genes coding for *Nrf2*, kelch-like ECH-associated protein 1 (*Keap1*), superoxide dismutase 1 (*SOD1*), *CAT*, *GSH-Px*, glutathione peroxidase (*GST*), heme oxygenase-1 (*HO-1*), NAD(P)H/ quinone dehydrogenase 1 (*NQO1*), and *β-actin* (reference gene) were quantified via real-time PCR with the 7500 real-time PCR detection system and Hieff^®^ qPCR SYBR^®^ Green Master Mix (Yeasen, Shanghai, China). The PCR mixture contained 10 μL of Hieff^®^ qPCR SYBR^®^ Green Master Mix, 1 μL, each, of the forward and reverse primers, 6 μL ddH2O, and 2 μL of cDNA sample. The thermal cycling conditions were as follows: 95 °C for 5 min, followed by 40 cycles of 95 °C for 10 s, and 56 °C for 20 s, and 72 °C for 20 s. All primers are presented in Table 2. The relative quantities of mRNA were calculated according to the 2^−ΔΔCt^ method [18].

### 2.6. Statistical Analysis

The general linear model was applied (Yij = µ + di + εij; Yij: the observation, µ: the general mean, di: the treatment effect, εij: the random error) and all data were subjected to one-way ANOVA by SPSS 20.0 (SPSS, Chicago, IL, USA) followed by Tukey’s multiple range tests with each bird as an experimental unit. All data are expressed as mean values with a standard error of means (SEM). Statistical significance was declared at *p* < 0.05.

## 3. Results

### 3.1. Carcass Traits

AHS treatment had no effect on slaughter percentage, semi-eviscerated carcass percentage, leg muscle yield, or breast muscle yield (*p* > 0.05) but resulted in a lower (*p* < 0.05) eviscerated carcass percentage (Table 3). The AHS + EGCG treatment had no effect on slaughter percentage, semi-eviscerated carcass percentage, leg muscle yield, or breast muscle yield of broilers (*p* > 0.05) but significantly increased (*p* < 0.05) the eviscerated carcass percentage compared with the AHS group. There were no statistically significant differences between CON and AHS + EGCG groups.

### 3.2. Meat Quality and Muscle Lactic Acid and Glycogen Contents

**Figure 1:**
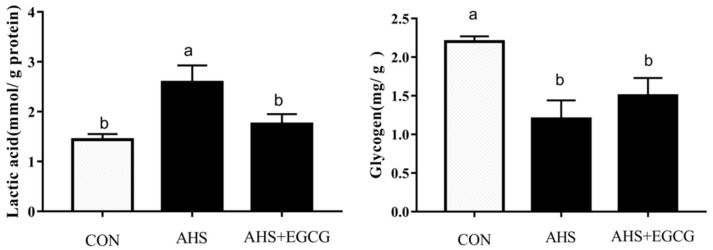
Effect of EGCG on lactic acid and glycogen contents in the muscle of broilers subjected to acute heat stress. ^a,b^ Values without common superscripts in the same row differ significantly (*p* < 0.05). CON, control group; AHS, acute heat stress group; AHS + EGCG, acute heat stress + epigallocatechin gallate group.

AHS had no effect on pH_45min_, cooking loss, shear force, b*, or L*_45min_ (*p* > 0.05) but resulted in higher (*p* < 0.05) L*_24h_, drip loss, and muscle lactic acid content and lower (*p* < 0.05) pH_24h_, a*, and muscle glycogen content (Table 4 and Figure 1). The AHS + EGCG group had lower (*p* < 0.05) L*_24h_, drip loss, and muscle lactic acid content and greater (*p* < 0.05) pH_24h_, a* compared with the AHS group. There were no statistically significant differences between CON and AHS + EGCG groups.

### 3.3. The Activities of Antioxidant Enzyme and the Contents of MDA and PC in Muscle

There were significant decreases (*p* < 0.05) in the T-SOD activity and the ratio of T-SOD/MDA and GSH-Px/MDA but significant increases (*p* < 0.05) in the MDA concentration of the breast muscle after AHS treatment (Table 5). Compared with the AHS group, AHS + EGCG treatment significantly increased (*p* < 0.05) T-SOD activity, the ratio of T-SOD/MDA, and decreased (*p* < 0.05) the MDA concentration. There were no statistically significant differences between CON and AHS + EGCG groups.

### 3.4. Expression of Genes Related to Nrf2 Signaling Pathway

The expression levels of *Nrf2*, *CAT*, and *NQO1* were downregulated (*p* < 0.05), while *Keap1* expression was upregulated (*p* < 0.05) by AHS treatment (Table 6). Compared with the AHS group, the AHS + EGCG group had upregulated (*p* < 0.05) *Nrf2* and *NQO1* expression levels and markedly downregulated (*p* < 0.05) *Keap1* expression. There were no statistically significant differences between CON and AHS + EGCG groups.

## 4. Discussion

EGCG is the most active substance in tea polyphenols and has developmental value and excellent biological activities, including anticancer, antiviral, and anti-inflammatory activities [19,20,21]. Research has shown that, in the absence of selenium, EGCG can activate different antioxidant defense systems in the liver of mice to maintain the redox balance in the body [22]. Moreover, a previous study found that supplemental EGCG promoted the expression of *Nrf2* in the intestinal mucosa of heat-stressed broilers [12]. Therefore, we infer that when broilers are subjected to HS, EGCG may be an inducer of the *Nrf2* signaling pathway to activate antioxidant defense systems for maintenance of the body’s redox balance.

Carcass traits are an important indicator of the economic performance of meat production. In this experiment, broilers undergoing AHS treatment at 33 °C for 12 h displayed a significant reduction in the eviscerated percentage. Smith [23] found that the dry matter contents of carcass parts (breast and thigh) were significantly decreased by elevated environmental temperature, but the contents of Na (breast) and K (back, breast, drumstick, and thigh) were significantly increased, indicating that elevated environmental temperature may decrease the organic matter contents in the carcass of broilers, which may partly explain why hot environmental temperature decreased the eviscerated carcass percentage. Excitingly, we found that adding EGCG to the diet can significantly reduce the negative effects of AHS on the eviscerated carcass percentage. However, further studies will be required to clarify the exact mechanism of action.

Meat quality of broilers is a complex trait that is determined by amino acid content, meat color, tenderness, pH, and water-holding capacity. AHS or chronic HS could negatively affect meat quality, causing decreased consumer acceptability and substantial economic losses [3,4,5,24]. Again, the results of the present study showed that meat quality was adversely affected by AHS, including increased drip loss and L*_24h_ and decreased pH_24h_ and a* values. In a previous study, it was found that chronic HS accelerated anaerobic glycolysis of muscle glycogen, leading to lactic acid accumulation and a reduced pH [25]. Similarly, the present study found that AHS decreased muscle glycogen content and increased lactic acid content. Other studies have shown that supplementing broiler diets with certain nutrients with antioxidant properties (mannan oligosaccharide, curcumin, glutamine) can significantly reduce the adverse effects caused by HS and improve the meat quality [8,9,26]. Based on the above analysis, we speculated that EGCG could prevent AHS-induced meat quality impairment of broilers, because of its antioxidant properties. As we expected, compared with the AHS group, the addition of EGCG to the diet did increase the pH_24h_ and a* values and decrease L*_24h_ and drip loss, indicating that EGCG can alleviate the AHS-induced reduction of meat quality. In addition, we also found that dietary EGCG tends to increase the content of muscle glycogen and significantly decrease the content of muscle lactic acid of broilers subjected to AHS and provides a reason for EGCG preventing AHS-impaired pH values in the breast muscle of broilers.

When free radical metabolism is abnormal, reactive oxygen species (ROS) accumulate in large quantities, which destroy the balance of the body antioxidant system and induce oxidative stress damage. Studies have shown that the adverse effects of HS on broilers are closely associated with excessive ROS production, which destroys biological macromolecules and causes cell damage [8]. The end product of the peroxidation reaction of free radicals acting on lipids in organisms is MDA. Generally, the MDA content reflects the degree of meat lipid peroxidation levels and relates to meat quality [27]. It has been demonstrated that AHS results in oxidative damage to the porcine skeletal muscle with increases in the MDA level [28]. Similarly, the present study confirmed that AHS increased MDA content in breast muscle of broilers, indicating decreased muscle antioxidant capacity and increased lipid peroxidation levels. Dietary supplementation with antioxidants, such as resveratrol [27] and curcumin [29], has been shown to be an effectual preventive measure against chronic HS-induced imbalances of lipid peroxidation, protein oxidation, and antioxidant systems. Moreover, Xue et al. [13] reported that EGCG significantly alleviated lipid peroxidation and oxidative stress in broilers subjected to chronic HS. As we expected, our results showed that supplemental EGCG effectively reduced the MDA content in the breast muscle of broilers subjected to AHS, indicating that EGCG can prevent the oxidative damage of the muscle caused by AHS.

The antioxidant enzyme activities in muscle, including T-SOD, GSH-Px, and CAT, are critical for meat quality at slaughter. In the resent study, the activity of T-SOD in muscle and the ratio of T-SOD/MDA and GSH-Px/MDA was decreased by AHS, indicating that the antioxidant balance of muscle was destroyed, which could partly explain why AHS decreased the meat quality and improved the MDA content in muscle of broilers. Previous studies found that the activities of CAT, SOD, and GSH-Px in serum, liver, and jejunum of broilers under HS were improved by EGCG [12,13]. The present study found that dietary EGCG supplementation had no significant effect on CAT and GSH-Px activities, but beneficially increased T-SOD activity and the ratio of T-SOD/MDA in the muscle of broilers subjected to AHS, indicating that the antioxidant capacity of muscle was improved to some extent, which may be the important factor that resulted in the improvement of meat quality of the broilers subjected to AHS.

Based on the above data, EGCG can improve the meat quality and muscle antioxidant capacity of broilers subjected to AHS, but its mechanism of action has not been studied. *Nrf2* mediates the transcription of many antioxidant enzyme genes with antioxidant response element (ARE), such as *HO-1*, *NQO1*, *SOD*, *CAT,* and *GSH-Px*, and plays an important role in cellular antioxidant function [30]. Usually, *Nrf2* is bound to Keap1 and exists in the cytoplasm. However, stress stimuli and some phytochemicals can destroy the cysteine residues of Keap1, resulting in a large accumulation of *Nrf2* in the cytoplasm, and then the unbound *Nrf2* translocates into the nucleus and binds to the promoter region of the ARE of many antioxidant genes, thereby initiating the transcription of the target genes [30,31]. Shanmugam et al. [32] observed that EGCG increased the expression of *Nrf2* and decreased the expression of *Keap1* of rats subjected to fluoride-induced oxidative stress. Previous studies in broilers showed that chronic HS significantly decreased jejunal *Nrf2* mRNA expression level [12] and liver *Nrf2* protein expression level [13], which could be significantly alleviated by dietary EGCG supplementation. Our present study found that the mRNA expression levels of *Nrf2*, *NQO1,* and *CAT* in breast muscle were decreased by AHS, while *keap1* mRNA level was increased, indicating that the AHS-induced decrease of muscle antioxidant capacity may be attributed to the abnormal expression of the *Nrf2* signaling pathway. Moreover, we firstly found that EGCG could not only significantly increase *Nrf2* and *NQO1* mRNA expression levels but also decrease *keap1* mRNA expression level in muscle of broilers under AHS, indicating that EGCG may activate the *Nrf2* signaling pathway, which may be the important reasons for the increased antioxidant capacity and reduced oxidative damage in muscle.

## 5. Conclusions

In summary, it was found that AHS induced a reduction of meat quality and increased oxidative damage in muscle of broilers. Dietary supplementation with EGCG (400 mg/kg) could improve meat quality and muscle antioxidant capacity of acute heat-stressed broilers, which may be associated with the activation of key antioxidant gene expressions in the *Nrf2* signaling pathway. These findings suggested that EGCG could be an effective additive to improve meat quality and muscle redox balance by regulating the *Nrf2* signaling pathway. However, further research is required to elucidate the exact mechanism of action.

## Figures and Tables

**Table 1:** Composition and nutrient levels of the basal diets.

Items	22–42 d	43–94 d
Ingredients (%)		
Corn	62.50	75.50
Soybean oil Corn protein meal	2.40 1.20	2.50 0.00
Soybean meal	30.33	23.77
_D, L_-Methionine	0.11	0.13
Salt	0.30	0.30
Choline chloride	0.15	0.15
CaHPO_4_·2H_2_O Limestone	1.62 1.16	1.32 1.10
Vitamin premix ^1^	0.03	0.03
Trace mineral premix ^2^	0.20	0.20
Total	100.00	100.00
Calculated nutrient levels		
Metabolizable energy, Mcal/kg	12.56	12.81
Crude protein, %	19.02	16.07
Lysine, %	0.99	0.85
Methionine + cysteine, %	0.72	0.65
Available phosphorus, % Calcium, %	0.40 0.91	0.35 0.80

^1^ The vitamin premix provided the following per kg of diet: vitamin A, 10,000 IU; vitamin D_3_, 3500 IU; vitamin E, 25 IU; thiamin, 2.00 mg; riboflavin, 6.00 mg; vitamin B_12_, 0.025 mg; vitamin K_3_, 2.5 mg; biotin,0.06 mg; folic acid, 1 mg; pantothenic acid, 10 mg; nicotinic acid, 40 mg. ^2^ The mineral premix provided the following per kg of diet: Cu, 8 mg; Fe, 100 mg; Zn, 100 mg; Mn, 120 mg; I, 0.7 mg; Se, 0.3 mg.

**Table 2:** Sequences of the primers used for the detection of gene expression levels.

Gene	Primer (5’-3’)	GenBank Number
*β-actin*	F: TGATATTGCTGCGCTCGTTG	NM_205518.1
	R: AACCATCACACCCTGATGTCTG	
*Nrf2*	F: TTCGCAGAGCACAGATACTTC	NM_205117.1
	R: TGGGTGGCTGAGTTTGATTAG	
*HO-1*	F: TGTCCCTCCACGAGTTCAAG	NM_205344.1
	R: CTCCGAGTTGCTGCCATAGAA	
*Keap1*	F: CTGCTGGAGTTCGCCTACAC	XM_025145847.1
	R: CACGCTGTCGATCTGGTATC	
*NQO1*	F: CTCCGAGTGCTTTGTCTACGA	NM_001277619.1
	R: ATGGCTGGCATCTCAAACC	
*SOD1*	F: GGAGTGGCAGAAGTAGAAATAGAAG	NM_205064.1
	R: AGGTCCAGCATTTCCAGTTAG	
*CAT*	F: GGCGTATGACCCTAGCAACA	NM_001031215.2
	R: TCTGATAATTGGCCACGCGA	
*GSH-Px*	F: ACGGCGCATCTTCCAAAG	NM_001277853.2
	R: TGTTCCCCCAACCATTTCTC	
*GST*	F: GGAAGCCATTTTAATGACAGA	XM_ 015284825.2
	R: TCCTTTAAAAGCCTGTAGCAGA	

*Nrf2,* nuclear factor erythroid 2–related 2; *HO-1*, heme oxygenase-1; *Keap1*, kelch-like ECH-associated protein 1; *NQO1*, NAD(P)H/quinone dehydrogenase 1; *SOD1*, superoxide dismutase 1; *CAT*, catalase; *GSH-Px*, glutathione peroxidase; *GST*, glutathione S-transferase.

**Table 3:** Effect of EGCG on carcass traits of broilers subjected to acute heat stress.

Items	CON	AHS	AHS + EGCG	SEM	*p*-Value
Slaughter percentage, %	87.6	85.0	87.0	0.560	0.518
Eviscerated carcass percentage, %	64.5 ^a^	59.9 ^b^	63.0 ^a^	0.641	0.004
Semi-eviscerated carcass percentage, %	79.0	78.0	79.2	0.410	0.498
Breast muscle yield, %	13.3	12.0	12.9	0.566	0.677
Leg muscle yield, %	22.0	22.6	22.4	0.280	0.731

^a,b^ Values without common superscripts in the same row differ significantly (*p* < 0.05). CON, control group; AHS, acute heat stress group; AHS + EGCG, acute heat stress + epigallocatechin gallate group.

**Table 4:** Effect of EGCG on meat quality in the broilers subjected to acute heat stress.

Items	CON	AHS	AHS + EGCG	SEM	*p*-Value
Drip loss, %	2.62 ^b^	3.60 ^a^	2.97 ^b^	0.142	0.005
Cooking loss, %	19.4	23.6	20.6	0.978	0.213
Shear force, N	23.9	28.8	24.8	1.88	0.543
pH_45min_	6.02	6.04	5.97	0.068	0.925
pH_24h_	5.55 ^a^	5.27 ^b^	5.66 ^a^	0.059	0.010
L*_45min_	48.3	51.8	49.6	0.751	0.146
a*_45min_	7.55 ^a^	5.89 ^b^	7.37 ^a^	0.298	0.031
b*_45min_	14.7	14.5	14.8	0.460	0.980
L*_24h_	49.5 ^b^	54.5 ^a^	50.0 ^b^	0.949	0.049
a*_24h_	8.97 ^a^	6.59 ^b^	8.58 ^a^	0.408	0.023
b*_24h_	14.4	15.0	13.4	0.580	0.547

^a,b^ Values without common superscripts in the same row differ significantly (*p* < 0.05). CON, control group; AHS, acute heat stress group; AHS + EGCG, acute heat stress + epigallocatechin gallate group.

**Table 5:** Effect of EGCG on the muscle antioxidant capacity, MDA, and PC contents of broilers subjected to acute heat stress.

Items	CON	AHS	AHS + EGCG	SEM	*p*-Value
MDA, nmol/mg protein	1.98 ^b^	2.92 ^a^	2.09 ^b^	0.177	0.049
PC, nmol/mg protein	18.7	25.7	22.0	1.62	0.219
T-SOD, U/mg protein	75.2 ^a^	61.6 ^b^	68.2 ^a^	2.34	0.045
CAT, U/mg protein	59.5	49.9	52.2	3.59	0.521
GSH-Px, U/mg protein	229	198	220	7.45	0.250
T-SOD/MDA	42.9 ^a^	21.6 ^b^	39.3 ^a^	3.81	0.038
CAT/MDA	28.0	18.8	25.0	2.18	0.229
GSH-Px/MDA	125 ^a^	70.9 ^b^	108 ^ab^	9.33	0.032

^a,b^ Values without common superscripts in the same row differ significantly (*p* < 0.05). CON, control group; AHS, acute heat stress group; AHS + EGCG, acute heat stress + epigallocatechin gallate group.

**Table 6:** Effect of EGCG on the expressions of *Nrf2* signaling pathway related genes in the muscle of broilers subjected to acute heat stress.

Items	CON	AHS	AHS + EGCG	SEM	*p*-Value
*Nrf2*	1.00 ^a^	0.759 ^b^	0.981 ^a^	0.042	0.024
*Keap1*	1.00 ^b^	1.469 ^a^	1.141 ^b^	0.067	0.011
*HO-1*	1.00	0.898	0.946	0.037	0.601
*NQO1*	1.00 ^a^	0.659 ^b^	0.903 ^a^	0.056	0.009
*CAT*	1.00 ^a^	0.749 ^b^	0.788 ^b^	0.046	0.027
*SOD1*	1.00	0.903	0.924	0.063	0.833
*GSH-Px*	1.00	0.891	0.968	0.026	0.199
*GST*	1.00	0.885	0.945	0.075	0.811

^a,b^ Values without common superscripts in the same row differ significant (*p* < 0.05). CON, control group; AHS, acute heat stress group; AHS + EGCG, acute heat stress + epigallocatechin gallate group.

## Data Availability

The data presented in this study are available on request from the corresponding author.
